# Frugal energy innovations for developing countries – a framework

**DOI:** 10.1002/gch2.1012

**Published:** 2016-09-30

**Authors:** Sini Numminen, Peter D. Lund

**Affiliations:** ^1^ School of Science Aalto University PO Box 15100 Espoo FI‐00076 Aalto Finland

**Keywords:** Developing countries, energy frugality, frugal innovations, frugality, renewable energy, resource‐scarcity, sustainable energy

## Abstract

Frugal innovations have recently emerged to feature low‐cost technologies and business innovations to serve consumers in emerging markets and improve their quality of life. Although the concept of frugality is well known, the present literature on frugal energy innovations, or energy frugality, is scarce, which could lead to overlooking its true characteristics. Therefore, we propose a framework for defining energy frugality based on a detailed analysis of several low‐cost sustainable energy technologies. The five‐criteria assessment method developed will help to identify potential frugal energy innovations and will increase the adoption of these technologies through better matching to local needs. Fuel‐efficient biomass cooking stoves, small‐scale photovoltaic systems, and pico‐grids are examples of such frugal energy technologies.

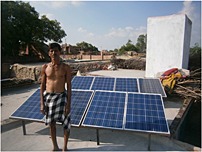

**Impact Box:** Over one billion people in developing countries live without any modern energy services. This study combats energy poverty through so‐called frugal energy innovations, which are affordable and sustainable energy innovations for poor people. Frugal energy innovations are based on effective use of local resources and skills, and renewable energy. Energy frugality is a new concept and has not yet been well defined. In this study, we provide a framework for frugal energy innovations to better help identify and further develop these kinds of technologies in the future. The potential for energy frugality is huge and urgent, considering the challenges faced by people in less‐developed countries. Indirectly, frugal energy products have a positive impact on climate change mitigation, food, water, and health issues as energy often helps to provide these services as well.

The solar photovoltaics (PV) pico‐grid system of Boond Engineering & Development Pvt Ltd. in Sathara village in North India. Photo: Boond (www.boond.net)

## Introduction

Energy is a necessity for progress and a better living standard, and it is crucial for human wellbeing (UN 2010). Global access to modern energy services is an important target set by many international organizations as well as national governments (Brew‐Hammond 2010; IEA 2011). However, 2.7 billion people still live without access to clean cooking facilities and 1.2 billion without access to electricity (IEA 2015). Nearly all of these people live either in sub‐Saharan African or Asian countries, mostly in rural areas. Introducing clean and efficient technologies that provide domestic energy services is clearly important to these regions, in particular for improved food preparation, space and water heating, lighting, small appliances, and communication.

Affordability influences access to energy services. Typically, the poorest quintiles of populations spend a substantial part of their income in fuels and energy services (Bacon et al. 2010) or other basic services (Prahalad 2005). In India for example, 11% of a household's income on average goes to fuels and energy services (Bacon et al. 2010). In Kenya or Uganda, where the Gross Domestic Product (GDP) per capita is less than $800, people in off‐grid areas may spend up to $80 a year on mobile phone charging services only (Manchester and Swan 2013). In comparison, EU households spend around 4% of their income on energy (Gerstberger and Yaneva 2013).

The lack of modern energy services often leads to using polluting alternatives, such as kerosene, or using inefficiently local energy sources, such as fuel wood. For example, solid biomass in different forms is still the main energy source for cooking and heating in Sub‐Saharan Africa and South Asian regions, but its use is inefficient causing serious health problems and such environmental damage as deforestation (Sesan 2012).

Clearly, introducing modern energy technologies could significantly improve the situation (Jannuzzi and Goldemberg 2012). Local renewable electricity production, such as photovoltaics (PV) or micro‐grids, is becoming viable options in developing countries (REN21 2015). Unfortunately, the adoption of different sustainable energy technologies still remains modest, calling for intensified efforts to improve the situation (IEA and World Bank 2015). One of the reasons for slow adoption may be insufficient understanding of local cultures and their institutional capacities for a pervasive adoption of new technologies (Murphy 2001; Carr 1985).

In response, several technology movements have historically been introduced along with industrialization in developing countries. The *appropriate* technology movement (Pattnaik and Dhal 2015) emphasized adjusting imported technologies into the local social environment. The *intermediate* technology movement highlighted the importance of small entrepreneurship in the transition (Schumacher 1974). When introducing modern energy services to poor communities, involvement of local entrepreneurship may better help to understand local consumer's preferences and adjust imported technology to local needs (Hansen et al. 2014).

Recently, frugal innovations have emerged to address the accessibility of new technologies in developing countries, in particular because frugal technologies have a lower purchase price than alternatives. These types of innovations involve small entrepreneurs or larger companies that succeed in overcoming the poverty gap and the lack of material resources, as well as several other practical problems in providing novel innovative solutions or services to people at the bottom of the economic pyramid (Radjou et al. 2012). Economic constraints are, in particular, tied to the low purchasing power of consumers and the technical constraints to insufficient infrastructures. For example, the lack of reliable access to grid electricity in an Indian town motivated the development of a cheap clay fridge for food storage, which works without electricity (Praceus 2014).

The academic literature on frugal innovation is still limited, and it has mainly been discussed within the business regime. Scientific literature on frugal innovations in providing energy services is also very scarce and often focuses on case studies only (Levänen et al. 2015). The question of environmental sustainability, which is always important in the case of energy, may not have received adequate attention in this context (Brem and Ivens 2013); although, several examples demonstrate that the technologies chosen may fulfill the sustainability criteria (Basu et al. 2013).

Because of the importance of improved energy services to poor countries on the one hand, and the lack of understanding of the theoretical and practical significance of frugal innovations for energy on the other hand, this paper aims to better conceptualize frugal innovations for the energy sector including criteria for defining and characterizing such innovations. To our knowledge, such an analysis has not yet been performed. The kind of systematic approach proposed here could be beneficial for the future development and deployment of sustainable energy technologies for less developed countries, in particular to more effectively harness local resources, skills, and labor. Also, the adoption of technologies that are better connected to local know‐how and conditions could address the sustainability question more efficiently than when just applying imported technology.

The paper is structured as follows. In Section 2, we briefly overview the extant literature on frugal innovations. In Section 3, we review frugal innovations in the energy sector to understand their characteristics, and based on this, sketch a framework for frugal energy innovations. In Section 4, we suggest a more detailed set of criteria for their definition. The paper ends with a discussion and conclusions.

## Frugal and Other Resource‐Constrained Innovation Concepts

To understand energy frugality or frugal innovations in energy, we first look at the extant literature on frugal innovations. Mainly studied in business studies and product design, a frugal innovation refers to simple but competitive innovations that have gained a breakthrough status in an emerging market among consumers with a low purchasing power (Radjou et al. 2012). Well‐known examples of frugal innovations can be found among health care or telecommunications products, for example, the portable electrocardiogram by General Electric or the robust Nokia 1100 mobile phone, which were made affordable through simplified product architectures. The Nokia 1100 with a robust and low‐power design was sold for just $15–20 and was once the best‐selling mobile phone worldwide (Sehgal et al. 2010).

The core characteristics of a frugal innovation are engineering simplicity as the use of raw materials and other resources needs to be minimized, which results in lower manufacturing cost (Rao 2013). Product simplifications could lead to considerable energy savings as well; for example, simplified design and reduced size of an automated teller machine dropped the power consumption by 80% compared with a standard unit (Bound and Thornton 2012). Other important features of frugal technologies are robustness and durability, especially in products that are used in remote areas.

As the concept of frugal innovation is quite new, the theoretical framework for frugality may still need to be improved (Bhatti and Ventresca 2013) as there is sometimes conceptual overlap to other resource‐scarce innovations (Cunha et al. 2014; Soni and Krishnan 2014). Frugal innovations differentiate themselves often through some novel technical feature or business model compared with just a cheaper product (Zeschky et al. 2014). Sometimes, similar products named as frugal in emerging markets are found in industrialized countries but with different purpose, for example, the portable thermocouple fridge (Tiwari and Herstatt 2012). In this case, the technical novelty is not obvious, but instead, the purpose is which was fulfilling the cold storage needs of the people in India.

It is important to note that several successful frugal innovations reported have originated from large international or multinational companies such as Haier, Lenovo, Nokia, General Electric, Tata, and Renault‐Nissan. Initially, the concept of frugal engineering was raised by Renault stating that frugal innovations could be a great opportunity for successful business in emerging markets by integrating the resourceful mindset of ingenious Indian engineers into the product development processes (Sehgal et al. 2010; Radjou 2014). A frugal innovation initially designed for emerging markets may sometimes also find its way to industrialized countries, but then, it is called a reverse innovation (Govindarajan et al. 2012).

Resource‐scarce innovations created by poor people themselves in their own living surroundings resemble frugal innovations. Such innovations are often called indigenous innovations (Gupta 2006) or grass‐roots innovations (Kumar and Bhaduri 2014; Pattnaik and Dhal 2015). Common features of these are that local professional expertise is utilized to modify or improve the product, and they enable a service delivery at a much lower price. For example, using a bicycle for water pumping or constructing a wind turbine from recycled materials (Kamkwamba 2010).

Frugal innovations by definition serve the underserved populations in developing countries (Gupta 2011; Radjou et al. 2012), but only few analyses have been made on the societal appropriateness of different frugal technologies in the environments where they are being used (Levänen et al. 2015). This applies to energy as well.

## Analysis of Possible Frugal Innovations in Energy

Next, we analyze several low‐cost technology innovations in the energy sector to better understand their frugal characteristics and to provide evidence for defining the framework for frugal energy innovations later in Section 4.

We start by analyzing four cases in Table 1, which have been defined in literature as frugal energy innovations (strong evidence). Basing on this, we extract their common characteristics. Finally in Tables 2 and 3, we analyze further technology examples from the perspectives of sustainability and local appropriateness.

**Table 1:** Four cases of energy services defined as frugal innovations in literature.

Name	Description	Energy services provided	Reason for frugality	Reference	Innovator
Husk Power System (product)	Micro‐grid where the power is made of rice husk (Gupta et al. 2013)	Lighting, charging mobile phones and other appliances	Technical: Frugalized technology producing lower product manufacturing costs	(Bhatti and Ventresca 2012)	Local company
Mitticool (product)	Refrigerator made out of clay which functions without electricity. Based on the cooling effect of water evaporation (Praceus 2014)	Food storage	Lower product price (than electric fridges). Frugal energy use: no maintenance costs and zero energy consumption. Simple technology	(Rao 2013)	Private craftsman and entrepreneur
SELCO (company)	Social enterprise providing various small‐scale solar products for low‐income customers	Lighting, domestic hot water	Business: Novel energy business model allowing affordable energy services for the poor	(Radjou et al. 2012)	Local company
Boond LTD (company)	Energy access company providing various small‐scale renewable energy products for low‐income customers	Lighting, charging mobile phones and other appliances (pico‐grids). Food preparations	Business: Networked energy business model for sales and customer support in Indian villages	(Urpelainen and Yoon 2014)	Local company

**Table 2:** Examples of locally manufactured and frugally engineered technologies for household‐level energy use.

Name	Description	Energy services provided	Reference	Details for frugal product architecture	Relation to local energy use and the society	Innovator	Country
Cookit	Semi‐concentrating solar cooker ideal for off‐grid areas with high solar insolation	Food preparation	(SCInet 2015)	Simple and robust design: only cardboard, aluminum foil and glue required	Minimal costs. Local manufacturing possible without advanced skills and tools	Non‐profit organization	USA (international)
Energy made in Uganda	Locally manufacturable and serviceable robust solar home system	Lighting, charging mobile phones and other small appliances	(Barbosa and Petersen 2013)	Simple electronics and the PV system design	Project employs local people in manufacturing centers. The battery type chosen is widely available in Uganda	Collaborative project of a university and a community organization	US & UG
Barefoot college	Training semi‐literate villagers “solar mamas” from all around the world to manufacture, install and maintain solar lighting systems	Lighting, charging mobile phones and other small appliances	(Ashden Awards 2003); (Panda 2015)	Simple electronics and the PV system design	Depends on the success in setting up and running the manufacturing centers of “solar mamas” in their home countries	Non‐governmental organization (NGO)	IN & global
Liter of Light (Day)	Light source	Lighting	(World Habitat Awards 2014)	Robust product design. Needed is a PET bottle, water and some fastening materials	Minimal costs. Local manufacturing possible without advanced skills or tools. Suitable for informal settlements	NGO	PH
Open‐source wind turbine	Locally manufacturable wind turbine, several designs	Lighting. Electricity for small and medium‐size appliances	(Piggott 2001)	Simplified turbine design. Certain parts, such as wooden blades, can be made of locally available materials	Affordable electricity. Local manufacturing possible in workshops equipped with basic tools	Private innovator	UK & international
Thermo‐siphon solar water heater	Modular solar thermal water heater	Domestic hot water	(Weiss and Schwarzmüller 2001)	Solar thermal technologies are robust by nature: absorber is made of thin copper sheet metal, copper pipes and coated with solar varnish	Affordability: saves expensive electricity typically used for water heating in Zimbabwe. Local manufacturing possible in workshops equipped with basic tools	International research and development cooperation project	ZI & AT

**Table 3:** Examples of frugally designed biomass stoves.

Name	Description	Reference	Frugal architecture details	Relation with local energy uses and the society	Innovator	Country
Kenya Ceramic Jiko (KCJ) Stove	The most well‐known example of a fuel efficient biomass cooking stove	(Opole 1988)	Minimizing the stove ceramic liner size leads to a thrifty use of local raw material (clay). Claddings made of leftover metal parts from large industries	Significant savings in household biomass expenditure compared to open fire. Elements manufacturing and assembly by local small industries	Project by the Kenyan government and aid organizations	KN
Upesi stove	A simplified and more affordable version of the Kenya Ceramic Jiko, targeted for rural areas	(Flavin and Aeck 2004)	Further simplification by removal of few metal parts	More affordable purchase price (than KCJ), while the same price of the energy service (cooking)	Project by the Kenyan government, aid organizations and women's groups	KN
Toyola Energy Ltd “coalpot” stoves	Producing and selling fuel efficient charcoal stoves	(Ashden Awards 2011b)	Robust and simple design, similar to KCJ stove	Significant savings in household biomass expenditure. Elements manufacturing and assembly by local small industries	Small enterprise	GH
Thermoelectric cook stove “Nicaragua energy”	Cook stoves for impoverished communities of Nicaragua	(Horman et al. 2013)	Robust and simple design. Small thermoelectric couple improves the combustion efficiency	Affordable energy service (customers to save a month's salary on fuel in Nicaragua)	Product design project of a university	NI & US

**Figure 1:**
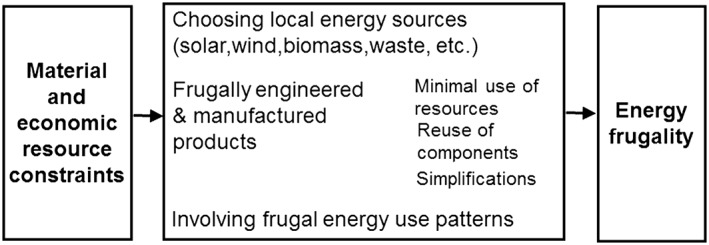
Process leading to energy frugality.

The analyses will follow a common framework shown in Figure 1 to identify energy frugality or features linked to it outgoing from the basic definition of frugal innovations.

Energy frugality as a concept has several implications as a result of the surrounding material and economic scarcities. Essentially, the lack of resources leads to designing technologies and products according to material‐saving frugal engineering principles, as presented by Rao (2013). In energy production, use of locally available energy sources is preferred, because they often are more affordable. In addition, economic constraints may result in thrifty and sparing uses of energy and in energy efficiency.

### Analysis of potential frugal energy innovations

We found in existing literature a few technological innovations in the energy sector that were defined as frugal innovations by the authors. These innovations, which are shown in Table 1, relate to food preparation and storage and household lighting. All examples in Table 1 are from India, which may be because the discourse on frugality is strongest there.

Bhatti and Ventresca (2012) defined Husk Power System in Table 1 as a frugal innovation because the technology can be produced with lower manufacturing costs because of frugal technical redesign, such as manufacturing the distribution grid insulation poles from bamboo instead of iron. The energy business has succeeded in bringing significantly more affordable electricity to the poor villages in India, which relied earlier on kerosene and diesel (Ashden Awards 2011a; Gupta et al. 2013).

The business dimension of energy frugality is highlighted by SELCO Solar Light Pvt Ltd. and Boond Engineering & Development Pvt Ltd., who deliver small‐scale solar electricity products for the poor in India, both being awarded for their achievements (Ashden Awards 2009; Economic Times 2014). Frugality relates here especially to the ability of these companies to carry out successful business models enabling low‐income consumers to purchase solar products. Both Boond (Urpelainen and Yoon 2014) and SELCO (Radjou et al. 2012) also arrange financial services for their customers with local banks.

**Figure 2:**
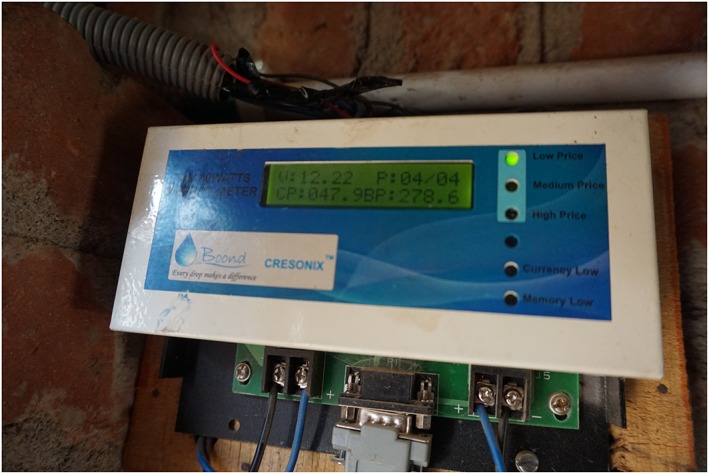
Energy meter of a pico‐grid is an example of a frugal solar component.

**Figure 3:**
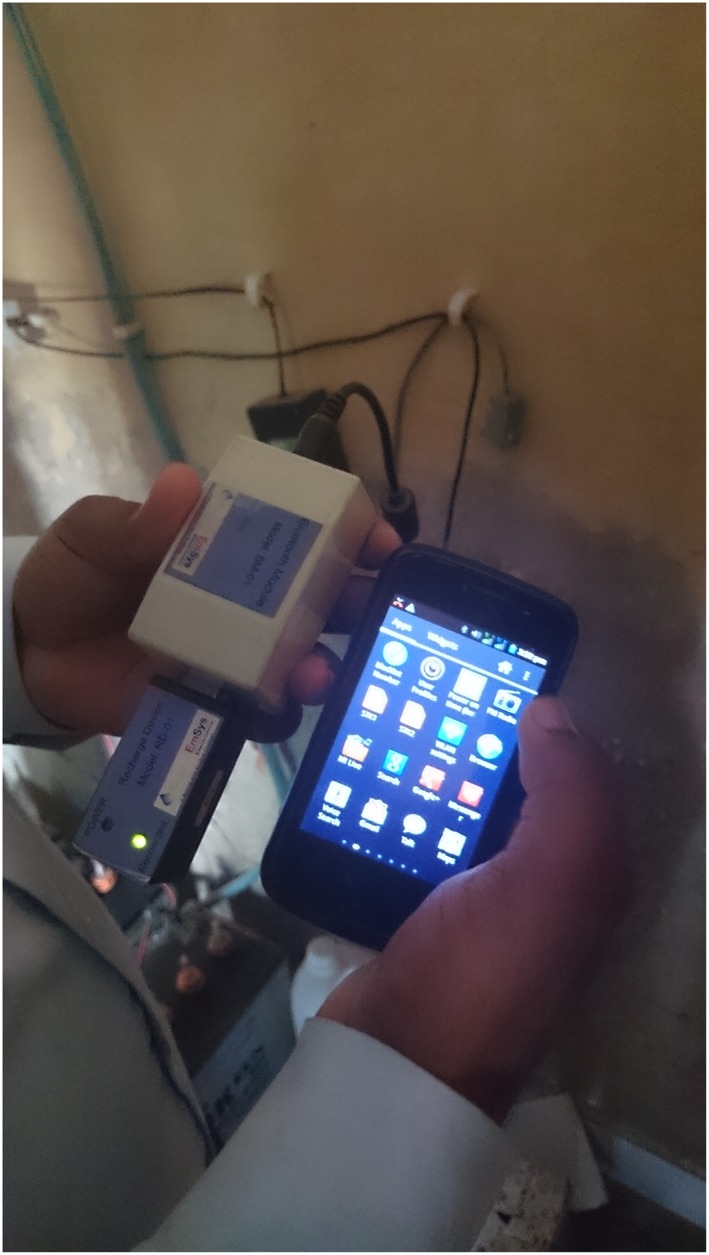
The pre‐payment dongle of a pico‐grid for charging the solar credits.

While Solar Home Systems for private households is a standard technology, also the technical product development work of these companies could deserve a closer look. Boond has developed another kind of innovation, the solar pico‐grid, which is a small micro‐grid that connects 5–50 households into a direct current distribution grid. The households are provided with a couple of LED light bulbs (à 3 W), a mobile phone charging device, and a fan and a TV in the case of larger grids (Kumar 2014). In every household, there is a frugally designed energy meter (Fig. 2) displaying the instantaneous consumption of energy and the availability of energy credits that are purchased in advance in a pre‐payment manner (Fig. 3).

Smart monitoring of the energy consumption on household level is an interesting feature of energy frugality. As a matter of fact, the prepaid model is an integral feature in many new micro‐grids in India (Fatima and Srivastava 2014) because it fits the unstable income of the consumers better than a post‐paid system.

Generally, the innovations in Table 1 help low‐income families to save in their energy expenditure, but the comparison with existing alternatives is not always straightforward as shown in the following. For example, the price of electricity from a solar pico‐grid (D'Agostino et al. 2016) can be compared with the costs of kerosene (Jain and Ramji 2016), if lighting is the main purpose. A customer in Uttar Pradesh, India, is typically charged 100 INR/kWh (1 INR = $0.015) for the pico‐grid electricity, which leads to a monthly bill of 140 INR for the lighting and mobile phone charging service (including three lamps). A simple payback analysis based on the investment cost (D'Agostino et al. 2016) and a 5‐years cycle yields a monthly cost of 158 INR. For comparison, with kerosene lamps (5 h/day), using the 2015 kerosene prices and accounting for the government subsidies gives a monthly cost of 118–148 INR (Jain and Ramji 2016). In reality, many rural areas in India do not enjoy of subsidized kerosene prices because of leakages in the national distribution system (Rao 2012), which would lead up to 175–250 INR.

Another difficulty in the comparison arises from the different service levels; for example, LED light bulbs provide significantly higher illuminance level than kerosene lamps (Mills 2016); that is, two LED lamps could possibly offer the same service as three kerosene lamps. In addition, the pico‐grid provides other services such as mobile phone charging. It also avoids health hazards typical to kerosene burning. Accounting for all these benefits and the previous price comparison speaks for the solar pico‐grid as an economic alternative.

Mainly because of the small size of the systems provided, technologies in Table 1 enable just basic energy services for lighting or mobile phone charging and not energy‐intensive services such as cooking. The sizing is also a trade‐off between income and service costs. In the pico‐grid, the instantaneous power per household is limited to 30 W, which is a result of the delicate optimization of the small energy system. Also other kinds of demand‐side management strategies have been implemented, for example, limiting the power availability to evenings (Boait 2014), a feature of micro‐grids by another solar entrepreneur, Mera Gao Power, whose customers are among the most marginalized in northern India (Urpelainen 2016). Energy frugality may not only be about an energy producing technology, but it could also enhance lower energy consumption both in the supply and demand side.

### Analysis of low‐cost and local energy innovations

Next, we picked six further cases of sustainable energy innovations used in emerging countries, not all of them defined as frugal technologies in the documentation, but which may include frugal aspects. We chose technologies for household‐level energy use from different countries based on two important criteria for frugal energy technologies: 1) energy delivery at an affordable price and 2) technology design based on frugal product design principles. Also, the technologies should have been successfully employed locally. However, we excluded typical community development projects such as micro‐hydro power as well as pure import of technology. Private grass‐root innovations were also left out because of lacking technical information. We restrict ourselves to renewable energy sources, which have a strong link to sustainable development.

The list of energy innovations chosen is shown in Table 2. We observed that the technical simplicity often enables local manufacturing, either in workshops (wind turbines and solar systems) or even at home (small lighting units and solar cookers). In some cases, a couple of components and parts need to be ordered from outside; but generally, the designs rely on standardized and low‐cost components that are available nearly all around the world (e.g., PET bottles, aluminum foil, basic electronic components, and cardboard), which has been found characteristic to frugally engineered products (Rao 2013). The panel style solar cooker (SCInet 2015) and the simple light source (World Habitat Awards 2014) can be manufactured with only a couple of dollars all around the world. After the initial investment, the energy service is available for nearly zero costs.

Energy made in Uganda is a project by the Frugal Innovation Lab of Santa Clara University for training of local people to manufacture and maintain simple solar home systems, thus targeting the affordability and locality of energy services (Barbosa and Petersen 2013; Energy Made in Uganda 2016). Barefoot College is another example of an initiative to educate and employ people in developing countries to manufacture small solar PV systems (Ashden Awards 2003). The specific potential of these two initiatives is that they do not only offer a technology but also involve the long‐term technical sustainability aspect. Lacking maintenance infrastructures and the lack of spare parts and the consequent system failures have been found to be a widespread problem with small PV systems in developing countries (Díaz et al. 2013), (Nieuwenhout et al. 2001), (Kumar et al. 2000). Strengthening of local manufacturing capabilities may respond to this.

As a matter of fact, the so‐called do‐it‐yourself philosophies are connected to frugal innovation (Reardon 2013; Banerjee 2015). Wind turbine blades, for example, can be made out of local wood material, if glass fiber is not available (Latoufis et al. 2015). Locally manufactured technologies, such as wind turbines, could actually have a considerable potential for the local economies in low‐income countries (Leary et al. 2012) linked to local employment and education. Often, a basic level of education only is required to manufacture frugal energy technologies.

As bioenergy in different forms is readily available in most developing countries (Karekezi 2002) and cooking is a major energy service, we also analyzed a set of biomass stoves to understand their possible frugal aspects. Table 3 lists four case stoves chosen. By nature, the stoves are simple and based on local technology, which could link them to frugal energy innovations. Some of the stoves could be even manufactured in workshops (Opole 1988); also, very energy‐efficient stoves have been developed, for example, the Kenyan Ceramic Jiko could save up to 50% of the fuel compared with open fire (Kammen 2000).

Frugality, as described in Figure 1, is demonstrated by these stoves both at the product design side as well as at the user side. Manufacturing of the KCJ and Upesi stoves can be achieved with local and recycled materials; the stove ceramic liner sizes are designed so that only the minimum amount of clay material is needed for insulation (Opole 1988). Initially, discarded metal drums from Nairobi's industries have been used as the metal claddings. At the energy user side, the stoves significantly contribute to the fuel saving and hereby to the reduction in household energy expenditures.

## Setting Criteria for Frugal Energy

Based on the analyses in the previous Section, we next elaborate a proposal for key criteria to define frugal energy technology to provide affordable and sustainable energy for low‐income households in developing countries.

Starting with the product architecture, a frugal energy technology is manufactured with minimal use of resources, and the technical design contains simplifications either in component or system levels. This should enforce that a more affordable product will result. The product design also incorporates inherent functionality (Rao 2013). The durability of the technology is a quality issue that needs special consideration, as the up‐front investments and payback times may be considerable to people in poverty. Therefore, a rugged and persistent design (Basu et al. 2013) will be important to avoid a too‐short product lifetime (Ashden Awards 2011c).

The organizational questions related to operations and maintenance are important as well. The installation, operation, and maintenance of frugal power systems may require skilled personnel. For example in the case of PV systems, although the PV panel is almost maintenance‐free, the battery unit may require frequent maintenance to ensure long‐term user satisfaction. Involving local skills for these kinds of services means local employment.

An important criterion to be set relates to sustainability, both in social and environmental contexts. These aspects have been highlighted less in the existing literature on frugal innovations. A sustainable energy solution makes use of the local and renewable energy sources and is also socially sustainable regarding the local culture and its habits in energy use preferences. For example, introducing a modern energy supply system may not automatically make families willing to switch away from traditional cooking (Murphy 2001).

Table 4 summarizes these criteria for frugal innovation in energy. Curiously, frugal energy technologies also demonstrate aspects of energy efficiency from the end user's side. Frugal ways of using energy mean modest output levels, and they mean a variety of ways for energy conservation, such as temporal limitations of power availability at times of energy shortage. The connection of energy frugality with energy efficiency shall be subject to further studies.

**Table 4:** Criteria for frugal and sustainable technologies providing energy services.

Main criteria	Comments
Frugal design and manufacturing	Minimal use of resources
	Simplified design
	Durability
Affordability	Low‐cost product
	Provision of a more affordable energy service
Local appropriateness	Socially fit for local user preferences
	Skills available for operation and maintenance
Environmental sustainability	Use of local renewable energy sources
	Recycled materials
	Avoiding harmful substances
Frugal energy use pattern	Efficient energy use and behavior
	Modest energy output level

## Discussion and Conclusions

Frugal innovations try to overcome the poverty gap by providing affordable products and services for people in developing countries and emerging economies. In this paper, we discuss the concept of frugal energy innovations and present a framework for defining these. We employed a two‐stage analysis of frugal technologies providing small‐scale energy services for low‐income consumers. First, a set of energy service technologies that have already been characterized as frugal innovations was investigated. It was found that the key frugality aspects were affordable energy services and a simplified technology design. Because of scarcity of literature on frugal innovations, the second set of examples was collected on affordable and sustainable energy technologies. Finally, a set of criteria for frugal and sustainable energy innovation was elaborated.

Fuel‐efficient biomass cooking stoves, small‐scale PV systems, pico‐grids, and other locally manufactured technologies are good examples of frugal energy technologies. The affordability is a central criterion for energy frugality, and it should preferably be viewed at the energy service level as technology may provide a service to a larger community than just for a single household, and in long‐term. Affordability differs from the pure price of technology, which because of lower manufacturing costs of a frugal innovation directly lead to a lower purchasing price and thus better accessibility. Finally, we proposed a five‐criteria framework to define energy frugality, covering affordability, frugal engineering, frugal energy use pattern, local appropriateness, and sustainability.

By definition, a frugal innovation addresses the needs of the poor. As frugal energy innovations often involve quite low power levels targeted to satisfy the basic needs only, for example, through off‐grid and stand‐alone energy applications, an important question will be if these innovations could become mainstream energy options or would they rather represent temporary solutions only. Technically the scale‐up would be easy, including direct current micro‐grids that can be enlarged to provide power for additional households and hamlets in the nearby regions (Madduri et al. 2013). Micro‐grids, in general, are becoming an important energy solution in remote areas in many developing countries because they can complement severe gaps in national power distribution systems (Palit and Bandyopadhyay 2016). For the energy service companies, however, the most modest power consumption levels of the cost‐conscious low‐income customers make it a challenging business (D'Agostino et al. 2016). Therefore, reaching large‐scale impact with the smallest off‐grid PV systems may be difficult without external financial support.

Recognizing that the existing literature of frugal energy innovations is very limited, future work on analyzing them could be well motivated, as these may include new information on frugality and functionality of technologies. Energy may also indirectly be present in other fields such as in agriculture or telecommunications, which could offer interesting implications for energy frugality, and may thus also deserve a closer look.

## Conflict of Interest

The authors declare no conflict of interest.
